# CCR7/dendritic cell axis mediates early bacterial dissemination in *Orientia tsutsugamushi*-infected mice

**DOI:** 10.3389/fimmu.2022.1061031

**Published:** 2022-12-22

**Authors:** Yuejin Liang, Hui Wang, Casey Gonzales, Joseph Thiriot, Piyanate Sunyakumthorn, Peter C. Melby, Jiaren Sun, Lynn Soong

**Affiliations:** ^1^ Department of Microbiology and Immunology, University of Texas Medical Branch, Galveston, TX, United States; ^2^ Institute for Human Infections and Immunity, University of Texas Medical Branch, Galveston, TX, United States; ^3^ Department of Pathology, University of Texas Medical Branch, Galveston, TX, United States; ^4^ Department of Veterinary Medicine, United States Army Medical Directorate, Armed Forces Research Institute of Medical Sciences (USAMD-AFRIMS), Bangkok, Thailand; ^5^ Department of Internal Medicine, University of Texas Medical Branch, Galveston, TX, United States

**Keywords:** CCR7, neutrophils, skin, draining lymph node, scrub tyhus, dendritic cells, bacterial dissemination, Orientia tsutaugmsushi

## Abstract

Scrub typhus is a life-threatening zoonosis caused by the obligate intracellular bacterium *Orientia tsutsugamushi* (*Ot*) that is transmitted by the infected larvae of trombiculid mites. However, the mechanism by which *Ot* disseminates from the bite site to visceral organs remains unclear; host innate immunity against bacterial dissemination and replication during early infection is poorly understood. In this study, by using an intradermal infection mouse model and fluorescent probe-labeled *Ot*, we assessed the dynamic pattern of innate immune cell responses at the inoculation site. We found that neutrophils were the first responders to *Ot* infection and migrated into the skin for bacterial uptake. *Ot* infection greatly induced neutrophil activation, and *Ot*-neutrophil interaction remarkably promoted cell death both *in vitro* and *in vivo*. Depletion of neutrophils did not alter bacterial dissemination in mice, as evidenced by similar bacterial burdens in the skin and draining lymph nodes (dLN) at day 3, as well as in the lungs and brains at day 14, as compared to the control mice. Instead, dendritic cells (DCs) and macrophages played a role as a Trojan horse and transmitted *Ot* from the skin into dLN. Importantly, the absence of homing receptor CCR7 or neutralization of its ligand, CCL21, significantly impaired DC migration, resulting in reduced bacterial burdens in dLN. Taken together, our study sheds light on a CCR7/dendritic cell-mediated mechanism of early *Ot* dissemination and provides new insights into therapeutic and vaccine development strategies for scrub typhus.

## Introduction

*Orientia tsutsugamushi* (*Ot*) is an obligate intracellular bacterium and the causative agent of scrub typhus. Scrub typhus is a mite-borne disease characterized by fever with a headache, suffused face, lymphadenopathy, skin lesion (eschar), and multi-organ involvement (1–3). Scrub typhus is geographically confined to an Asia Pacific region previously considered the “tsutsugamushi triangle”, where a billion people are at risk and nearly a million cases are reported every year (4–6). However, recent reports of *Ot* cases in areas previously thought free from scrub typhus, such as South America, raised the warning that *Ot* might be no longer restricted to the “tsutsugamushi triangle” (7). Patients who receive early antibiotic treatment (e.g., doxycycline) usually recover quickly, however, delayed diagnosis and neglected treatment of scrub typhus can result in severe complications including pneumonitis, acute respiratory distress syndrome, acute renal failure, myocarditis, and encephalitis (8). If not treated, the median mortality of scrub typhus is around 6%, with high mortality likely associated with central nervous system involvement and multi-organ failure (9). Currently, no effective vaccine is available for this neglected disease, partially due to the antigenic diversity among different *Ot* strains and the undefined roles of host immune responses in infection control versus disease pathogenesis.

*Ot* is spread among the natural rodent hosts and can transmit to people through bites of infected chiggers (larval mites) (10, 11). An eschar (a dark, scab-like region at the site of the chigger bite) can be found in some cases, often correlating with enlarged regional lymph nodes (12, 13). Examination of eschar skin biopsies from scrub typhus patients revealed that the *Ot* cellular tropism consists of host dendritic cells (DCs) and monocytes (10), which may be infected by *Ot* to promote bacterial dissemination from the dermis into lymphatic vessels (14). In addition, histological analysis of *Ot* in paraffin-embedded archived autopsy tissues revealed the localization of bacteria to endothelial cells of all peripheral organs, as well as in the central nervous system (11, 15, 16). Together, these studies suggest that the variety of target cells and immune activities elicited by *Ot* infection can be attributed to different tissue involvement and various stages of infection. Although we and others have uncovered the unique type 1-biased immunity and remarkable endothelial damage during severe *Ot* infection stages (16–23), early bacterial dissemination and local immune responses in the dermis and draining lymph nodes (dLN) are unclear.

Several animal models have been established by using a variety of infection routes and mouse strains to investigate *Ot* dissemination, pathogenicity, host protective/pathogenic immunity, and immunopathogenesis (24). Unlike intravenous and intraperitoneal infection, intradermal (i.d.) infection more closely mimics the natural infection by chigger bite and is considered a better model for studying the early dissemination of *Ot* and the related host immune responses (24–27). We previously established an i.d. inoculation model of scrub typhus by using C57BL/6j mice and reported the dynamic patterns of host Th1/2 immune responses, periphery organ immunopathogenesis and bacterial persistence, indicating that the i.d. infection mouse model can mimic systemic infection of *Ot* in humans (28). Using the i.d. inoculation model, Keller et al. also report increased bacterial loads in the dLN as early as day 3 post-infection, when bacteria were undetectable or very limited in major peripheral organs (26). This finding suggests that dLN might be a key component for *Ot* lymphatic dissemination from the skin inoculation site to internal target organs. However, the cellular and molecular mechanism(s) by which *Ot* transits into dLN is unknown. Uncovering this underlying mechanism for *Ot* dissemination will provide new insights into therapeutic strategies and vaccine development.

The difficulty in studying obligate intracellular bacteria is partially due to their genetic intractability (29). To our knowledge, no genetic toolbox has been validated for *Ot* to date (29). In this study, we tracked *Ot n vivo* by using the carboxyfluorescein succinimidyl ester (CFSE) labelling method (30) and analyzed the skin immune cell pattern at various time-points by multicolor flow cytometry. Our results demonstrated neutrophils as major phagocytes for engulfing bacteria at the inoculation site of infection. However, neutrophils might play a dispensable role in bacterial dissemination and replication, as they underwent *Ot*-induced apoptosis. Further analysis demonstrated that DCs and macrophages might be the Trojan horse for bacterial dissemination from the skin inoculation site to dLN *via* a CCR7/CCL21-mediated mechanism.

## Materials and methods

### Animals, infection and treatment

Female C57BL/6 (#000664) and CCR7^-/-^ mice (#006621) (31) were purchased from Jackson Laboratory. Mice were maintained under specific pathogen-free conditions and used at 8-10 weeks of age, following protocols approved by the Institutional Animal Care and Use Committee (protocol # 2101001) at the University of Texas Medical Branch (UTMB) in Galveston, TX. All mouse infection studies were performed in the ABSL3 facility in the Galveston National Laboratory located at UTMB; all tissue processing and analysis procedures were performed in the BSL3 or BSL2 facilities. All procedures were approved by the Institutional Animal Care and Use Committee (IACUC) and the Institutional Biosafety Committee, in accordance with Guidelines for Biosafety in Microbiological and Biomedical Laboratories. UTMB operates to comply with the United States Department of Agriculture (USDA) Animal Welfare Act (Public Law 89–544), the Health Research Extension Act of 1985 (Public Law 99–158), the Public Health Service Policy on Humane Care and Use of Laboratory Animals, and the NAS Guide for the Care and Use of Laboratory Animals (ISBN-13). UTMB is a registered Research Facility under the Animal Welfare Act and has a current assurance on file with the Office of Laboratory Animal Welfare, in compliance with NIH Policy.

Mice were inoculated with *Ot* Karp strain (4 × 10^3^ focus forming units (FFU), 10 -12 µL volume) or phosphate buffered saline (PBS, mock control) in the dermis of the lateral ears by using a 30 G-needle and 25-μl Hamilton syringe (Hamilton Company, Reno, NV) (28). Mice were monitored daily for weight loss, signs of disease, and survival. Some mice were intraperitoneally injected with anti-Ly6G, anti-Gr-1 or anti-Ly6G/6C (clone#1A8, RB6-8C5 and NIMP-R14, respectively; 250 µg/mouse, BioxCell, Lebanon, NH) at day -1, 0, 1, and 2 post-infection. Control mice were treated with Rat IgG (250 µg/mouse). For blocking local chemokine signaling, mice were i.d. injected with anti-CCL19 (2 µg, #AF880, R&D, Minneapolis, MN), anti-CCL21 (2 µg, #AF457, R&D, Minneapolis, MN) or both (2 µg/each, 4 µg total) at 1 day prior to infection. Goat IgG (#Ab-108, R&D) was used as a control or supplemented to the equal amount of antibody (4 µg total) per injection. At indicated time-points, mice were euthanized by CO_2_ inhalation and tissues were harvested for further analysis.

### Bacterial preparation and CFSE labelling

*Ot* was prepared from heavily (80-100%) infected L929 cell cultures (22). Briefly, bacteria were inoculated onto confluent monolayers in T150 cell culture flasks and gently rocked for two hours at 37°C, at the end of which Minimum Essential Medium (MEM) with 10% fetal bovine serum, 100 units/mL of penicillin and 100 µg/mL of streptomycin were added. Cells were harvested by scraping at day 7 post-infection, re-suspended in MEM, and lysed using 0.5 mm glass beads by vortex for 1 min. The cell suspension was collected and centrifuged at 300×g for 10 min to pellet cell debris and glass beads. The supernatant from one T150 flask was further inoculated onto new monolayers of five T150 flasks. This process was repeated for a total of six passages. Cells from five flasks were pooled in a 50 mL conical tube with 20 mL medium and 5 mL glass beads. The conical tubes were gently vortexed at 10 sec intervals for 1 min to release the intracellular bacteria and placed on ice. The tubes were then centrifuged at 700×g to pellet cell debris, and the supernatant was collected in Oakridge high speed centrifugation bottles, followed by the centrifugation at 22,000×g for 45 min at 4°C to harvest bacteria. Sucrose-phosphate-glutamate buffer (0.218 M sucrose, 3.8 mM KH_2_PO_4_, 7.2 mM KH_2_PO_4_, 4.9 mM monosodium L-glutamic acid, pH 7.0) was used for preparing bacterial stocks, which were stored at -80°C (17, 32). The same lot of *Ot* Karp stocks was used for all experiments described in this study.

For inoculation of L929 by using DC lysates, we harvested dLN from 30 mice at day 2 post-infection and pooled them for the purification of CD11c^+^ population by using anti-CD11c magnetic beads (Miltenyi Biotec, San Jose, CA). CD11c^-^ cells were also collected as a control. We then lysed 2 × 10^6^ cells using glass beads, as we described above, and seeded cell lysates into L929 cells in T12.5 flasks. At day 7, L929 cells were harvested, and cell lysates were further seeded into new T12.5 flasks with L929 cells. At day 14, cells were collected for the measurement of bacterial burdens by qPCR.

The CFSE labelling of bacteria was performed according to a previous report (30). Briefly, bacterial stock was added into a 15 mL tube of 1 mL PBS. One microliter of CFSE (5 mM, Biolegend, San Diego, CA) was added into the tube, followed by a gentle vortex. Samples were incubated for 10 min at 4°C in the dark. After incubation, the remaining dye was quenched by adding 10-fold volume of MEM + 10% FBS and incubated for 5 mins. The labeled bacteria were pelleted at 22,000 × g for 15 min, washed once with cold PBS, and resuspended either by cold PBS for animal infection or MEM for *in vitro* cell infection.

### qRT-PCR

Cells were lysed with RLT lysis buffer for 10 min, and RNA was extracted using RNeasy Mini kits (Qiagen, Germantown, MD), followed by the synthesis of cDNA using an iScript Reverse Transcription kit (Bio-Rad, Hercules, CA). cDNA was amplified in a 10 μL reaction mixture containing 5 μL of iTaq SYBR Green Supermix (Bio-Rad, Hercules, CA) and 5 μM each of gene-specific forward and reverse primers. The PCR assays were denatured for 30 s at 95°C, followed by 40 cycles of 15 s at 95°C, and 60 s at 60°C, by utilizing the CFX96 Touch real-time PCR detection system (Bio- Rad, Hercules, CA). Relative quantitation of mRNA expression was calculated using the 2^−ΔΔCt^ method. The primers are listed in **Table S1**.

### Quantitative PCR for measuring bacterial loads

To determine bacterial burdens, tissues (e.g., skin, lung and brain) were collected and incubated with proteinase K at 56°C. DNA was then extracted using a DNeasy Blood & Tissue Kit (Qiagen, Germantown, MD) and used for qPCR assays, as previously described (23, 32). The 47 kDa gene was amplified using the primer pair OtsuF630 (5’-AACTGATTTTATTCAAACTAATGCTGCT-3’) and OtsuR747 (5’-TATGCCTGAGTAAGATACGTGAATGGAATT-3’) primers (IDT, Coralville, IA) and detected with the probe OtsuPr665 (5’-6FAM-TGGGTAGCTTTGGTGGACCGATGTTTAATCT-TAMRA) (Applied Biosystems, Foster City, CA) by SsoAdvanced Universal Probes Supermix (Bio-Rad, Hercules, CA). Bacterial loads were normalized to total nanogram (ng) of DNA per µL for the same samples. The copy number for the 47-kDa gene was determined by known concentrations of a control plasmid containing a single-copy insert of the gene. Gene copy numbers were determined *via* serial dilution (10-fold) of the *Ot* Karp 47-kDa plasmid.

### Neutrophil purification and *in vitro* infection

Bone marrow cells were collected from the tibia and femur of B6 mice, followed by incubation with Red Cell Lysis Buffer (Sigma, St. Louis, MO) for 5 min at room temperature to remove red blood cells. Neutrophils were purified by anti-Ly6G magnetic beads in LS columns of positive selection (Miltenyi Biotec, San Jose, CA) (33). The purity of neutrophils was confirmed by flow cytometry as ~96% (**Figure S1**). For cell infection, 5 × 10^5^ neutrophils were seeded in 12-well plates. After one-hour incubation in 37°C and 5% CO_2_, bacteria were added to the plate at MOI 10, followed by a gentle mixing by pipette and continuous culture in the incubator. At 4, 10 and 18 h post-infection, cells were harvested in 1.5 mL Eppendorf tubes by centrifugation (300×g, 10 min, 4°C) and lysed for 5 min using RLT buffer (Qiagen, Germantown, MD). The lysed samples were stored in -80°C until the use of RNA isolation.

### Bone marrow-derived DC generation and infection

Bone marrow-derived DCs were generated from B6 mice by cultivation with rGM-CSF (20 ng/ml), as described previously (34). Fresh GM-CSF-containing medium was added at days 3 and 6. DCs were harvested at day 8 and infected with *Ot* (MOI 10). Cells were collected at 24 and 48 h post-infection and analyzed for MHCII, CD80 and CD86 by flow cytometry. Bacterial loads were measured at 24, 48, 72 and 96 h post-infection by qPCR.

### Flow cytometry

For detection of infiltrated immune cells in the skin, each ear was collected in 1.5 mL Eppendorf tubes with 1 mL accutase (Biolegend, San Diego, CA) and cut with scissors for two min into small pieces. Minced tissues were loaded into Medicons and homogenized by a BD Mediamachine System (BD Biosciences, San Jose, CA) (23). Single-cell suspensions were prepared by passing cell homogenates through 70-µm cell strainers and treated with a Red Cell Lysis Buffer (Sigma, St. Louis, MO). Draining lymph nodes were also collected and passed through cell strainers for preparing single cell suspension. For surface marker analysis, leukocytes were stained with the Fixable Viability Dye (eFluor 506, Thermo Fisher Scientific, Waltham, MA) for live/dead cell discrimination, blocked with FcγR blocker, and incubated with fluorochrome-labeled antibodies (Abs) for 30 min. For apoptosis detection, cells were washed twice with Annexin V Binding Buffer (BD Bioscience, San Jose, CA), and stained with Annexin V. The fluorochrome-labeled antibodies were purchased from Thermo Fisher Scientific and Biolegend as below: APC-efluor 780 anti-CD45 (30-F11), Alexa Fluor 700 anti-CD11b (M1/70), APC anti-Ly6G (1A8), PE/Dazzle-594 anti-Ly6C (HK1.4), BV786 anti-CD24 (M1/69), BV650 anti-CCR2 (475301), PE anti-CD64 (X54-5/7.1), efluor450 anti-MHCII (M5/114.15.2), Percp-efluor710 anti-EpCAM (G8.8), PE-Cy7 anti-CD80 (16-10A1), APC anti-CD86 (GL-1), APC Annexin V, Fixable Viability Dye eFluor 506, and BUV395 anti-CD172a (P84). Cells were fixed in 2% paraformaldehyde overnight at 4°C before analysis. Data were collected by a BD LSR Fortessa and analyzed *via* FlowJo software version 10 (BD, Franklin Lakes, NJ).

### Immunofluorescent staining and fluorescent microscopy

Superficial cervical lymph nodes were removed and fixed in 4% paraformaldehyde (PFA) and embedded in Tissue-Tek OCT. Cryosections (6 µm) were fixed for 10 min in ice-cold acetone and rehydrated with PBS. For immunofluorescent staining, sections were permeabilized and blocked using 5% normal goat serum, 0.2% Triton X-100 in PBS for 1 h, followed by primary antibody incubation overnight at 4°C. The Alexa Fluor488 anti-mouse LYVE1 (ALY7, 1:500 dilution) was purchased from Thermo Fisher Scientific. The rabbit anti-*Orientia Karp* TSA56 polyclonal antibody (1:200 dilution) was generated and validated by Genscript (Piscataway, NJ). The Alexa Fluor555-conjugated anti-rabbit IgG (H+L), F(ab’)2 Fragment secondary antibody (1:500 dilution, Cell Signaling Technology, Danvers, MA) was used to stain the sections for 1 h at room temperature, followed by three washes with PBS. The TrueVIEW Autofluorescence Quenching Kit (Vector Laboratories, Newark, CA) was applied to diminish unwanted autofluorescence and the microscope slides were covered by using Fluoroshield Mounting Medium with DAPI (Abcam, Cambridge, MA). Images were acquired by an Olympus IX73 inverted microscope equipped with a color camera for light microscopy and with a monochrome camera to capture fluorescent images with standard wavelength filters. All images were captured under the same conditions of laser power, detector gain, and background offset.

### Statistical analysis

Data are presented as mean ± standard error of the mean. Differences between individual treatment and control groups were determined using a two-tailed, unpaired Student’s t-test. One-way ANOVA was used for multiple group comparisons, with Tukey’s *post hoc* for multiple comparisons among group means. Statistically significant values are referred to as *, *p <*0.05; **, *p <*0.01; ***, *p <*0.001; ****, *p <*0.0001; NS, no significant difference, respectively.

## Results

### Innate immune cell infiltration in the skin inoculation sites of *Ot*

**Figure 1:**
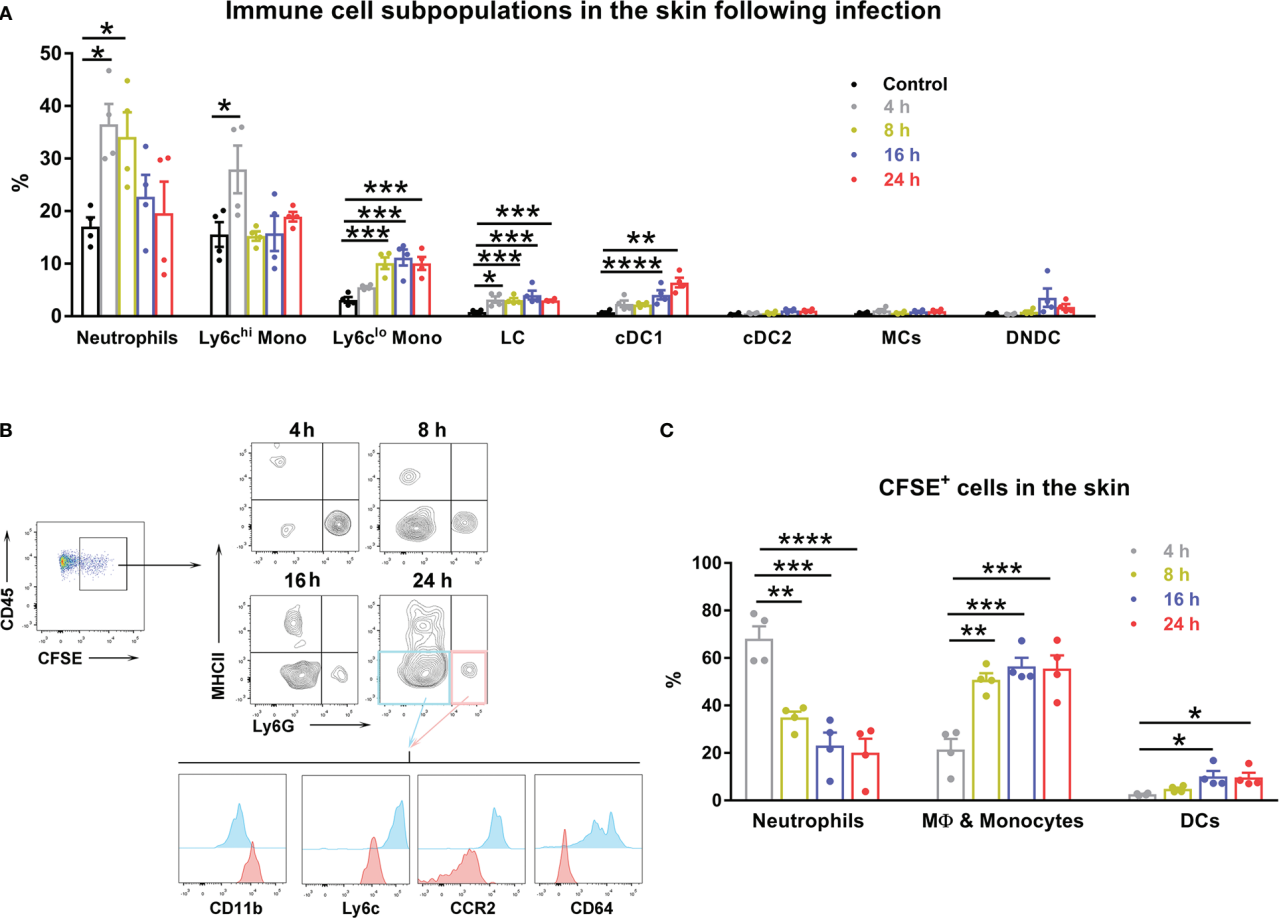
Innate immune cells displayed a dynamic pattern at the inoculation site of *Ot* infection. B6 mice (n=4/group) were intra-dermally (i.d.) infected with CFSE-labeled *Ot* (4×10^3^ FFU) in the ears. Skin tissue was harvested at indicated time-points, digested by collagenase, and prepared for single cell suspension. Control mice were euthanized immediately after injection. **(A)** The dynamic pattern of innate immune cell subpopulations infiltrating the inoculation site is displayed. **(B)** CD45^+^ and CFSE^+^ positive immune cells from infected mice were further characterized by immune cell types using surface markers. MHCII^+^ cells were considered antigen presenting cells including cDC1 (MHCII^hi^CD24^hi^CD64^-^EpCAM^-^), cDC2 (MHCII^hi^CD172a^hi^CD11b^+^CD64^-^EpCAM^-^), DN (double negative) DC (CD11b^-^MHCII^hi^CD24^-^CD64^-^EpCAM^-^), and Langerhans cells (MHCII^hi^CD24^hi^EpCAM^+^). Monocyte-derived inflammatory cells (MC) were identified as MHCII^+^CD11b^hi^CD64^hi^Ly6C^-^. MHCII^-^Ly6G^+^ cells (pink quadrant), which were also CD11b^hi^Ly6C^int^CCR2^int^CD64^-^, were characterized as neutrophils. MHCII^-^Ly6G^-^ cells (blue quadrant) were identified as macrophages and monocytes, as they were CD11b^int^Ly6C^+^CCR2^hi^CD64^+^. **(C)** The dynamic pattern of *Ot* infected-immune cells at the site of *Ot* inoculation 4 h post-infection is shown. Values are shown as mean ± SEM from single experiments and are representative of two independent experiments. A one-way ANOVA with a Tukey’s multiple comparisons test was used for statistical analysis. *, *p*<0.05; **, *p*<0.01; ***, *p <*0.001; ****, *p <*0.0001.

Scrub typhus is an acute febrile disease that occurs due to the accidental transmission of *Ot* through human skin by the forest dwelling vector, *Leptotrombidium* larva (1). Most studies of scrub typhus in animal models have adopted either intraperitoneal or intravenous inoculation of bacterial stocks (17, 21, 24, 32, 35); however, studies of host innate responses and bacterial dissemination in a natural route of infection are limited. By using an intradermal murine model of *Ot* infection that we established (28), we examined local dynamics of immune cell populations in the skin within the first 24 h of infection (**Figure 1A**). At 4 h post-infection, infiltrated cells detected in the inoculation site were predominantly neutrophils (~35%) and ly6C^hi^ monocytes (~28%); the percentages of these cells were decreased to 20% at 24 h. The proportions of Ly6C^lo^ monocytes, Langerhans cells (LC), and conventional type 1 dendritic cells (cDC1) were gradually increased following infection. Limited conventional type 2 dendritic cells (cDC2), monocyte-derived proinflammatory cells (MCs) and double negative DCs were observed during 24 h of infection.

To investigate bacterial dissemination from the skin to visceral organs, we first determined which cells were infected by *Ot* in the inoculation site. We i.d. injected CFSE-labeled bacteria into mouse ears, isolated skin immune cells at various time-points, and identified *Ot*-infected cells by flow cytometry. Our results showed that neutrophils (MHCII^-^Ly6G^hi^CD11b^hi^Ly6C^int^CCR2^lo^CD64^-^) and macrophages/monocytes (MHCII-Ly6G-CD11b^int^Ly6C^hi^CCR2^hi^CD64^+^) accounted for 70% and 20% of the infected cells at 4 h post-infection, respectively (**Figures 1B, C**). The proportion of infected neutrophils gradually declined to 20% at 24 h. In contrast, *Ot* infected more macrophages/monocytes (~60%) and DCs (~15%) at 24 h (**Figure 1C**). These results suggest that these subsets of myeloid cells may facilitate *Ot* dissemination from the skin to internal organs.

### *Ot* infection induced neutrophil activation and apoptosis

**Figure 2:**
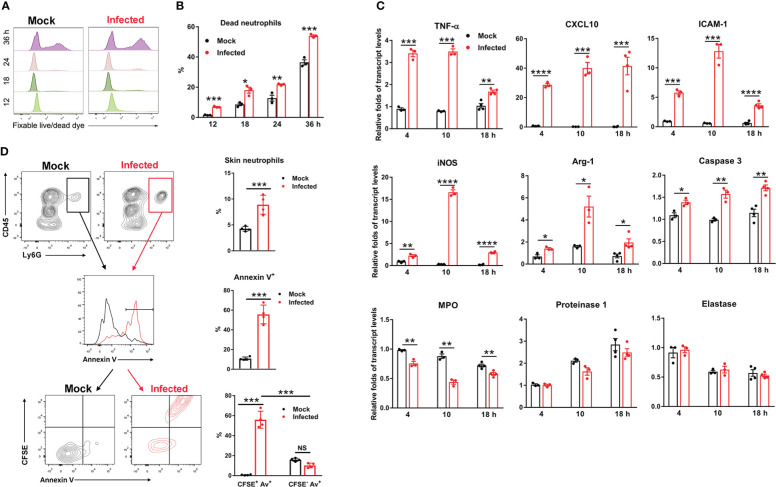
*Ot* infection triggered neutrophil activation and apoptosis. Bone marrow-derived neutrophils were prepared by using anti-Ly6G magnetic beads. The purity of BM neutrophils is 96% based on flow cytometric analysis. Cells were infected with *Ot* (MOI: 10) *in vitro* and harvested at indicated time-points. **(A)** The viability of cells was determined by using fixable live/dead viability dye. **(B)** The percentages of dead neutrophils at various timepoints were presented based on the flow cytometric analysis. **(C)** Cells were cultured *in vitro*, infected with *Ot* and harvested at 4, 10 and 18 hours (h) post-infection. The transcript levels of activation (TNF-α, CXCL10, ICAM-1, iNOS and Arg-1), apoptosis (Caspase-3) and granules (MPO, Proteinase-1 and Elastase) were detected using qRT-PCR. **(D)** B6 mice (n=4/group) were i.d. infected with CFSE-labeled *Ot* (4×10^3^ FFU) in the ears. Mock mice (n=4/group) were i.d. injected with PBS as a control. Skin tissues were harvested at 18 h, digested by collagenase for preparation of a single cell suspension, and then analyzed by flow cytometry. Neutrophils from mock (black) and infected (red) groups were first gated as CD45^+^Ly6G^+^ cells (left panel), and quantified (right panel). Neutrophils were further sorted for Annexin V expression on the cell surface (left panel), and quantified (right panel). Finally, neutrophils were sorted by CFSE expression (*Ot*-infected) and Annexin V expression (apoptosis) simultaneously (left panel), and quantified (right panel). Values are shown as mean ± SEM from single experiments and are representative of two independent experiments. A two-tailed Student’s t-test was used for the comparison of two groups. *, *p*<0.05; **, *p*<0.01; ***, *p <*0.001; ****, *p* < 0.0001.

Neutrophils are the most abundant leukocytes patrolling the body, and play a central role in host defense (36, 37); however, they can also contribute to pathogen dissemination, acting as a Trojan horse during infection with intracellular microbes (e.g., *Leishmania major* and *Brucella abortus*) (38–41). In the eschar biopsy of scrub typhus patients, neutrophils can form an intense zone of infiltration around the necrotic eschar center (10). Having demonstrated that neutrophils were the first responders and the major target of *Ot* infection at the inoculation site (**Figure 1**), we then examined the fate and function of these infected neutrophils during *Ot* infection. We infected bone marrow-derived neutrophils with *Ot in vitro* (MOI 10) and assessed cell viability by flow cytometry. We found that *Ot*-infected neutrophils displayed more cell death (~7%) at 12 h compared with the mocks (~2%) (**Figures 2A, B**). Cell death increased gradually at 18 and 24 h in both mock and infected groups, but infected cells displayed a two-fold higher death rate than mocks. At 36 h, more than half of neutrophils died by *Ot* infection, while the death rate in the mock group was less than 40% (**Figures 2A, B**). To determine neutrophil activation, we analyzed transcript levels of pro-inflammatory genes, including *TNF-α*, *CXCL10*, *ICAM-1* and *iNOS*. Our qRT-PCR results showed significantly increased expression of these genes at 4 h, with peak expression at 10 h (**Figure 2C**). High *CXCL10* expression levels were sustained at 18 h, while the expression of other markers (*TNF-α, ICAM-1* and *iNOS*) decreased. Arg-1, the enzyme for metabolizing arginine to ornithine and thereby reducing extracellular arginine at sites of infection (42), was slightly increased in infected cells at 4 h, followed by a 2.5-fold and one-fold upregulation at 10 and 18 h, respectively (**Figure 2C**). Consistent with our finding that *Ot* promoted neutrophil death (**Figures 2A, B**), we found elevated caspase-3 transcript levels in neutrophils with *Ot* infection at all examined time-points (**Figure 2C**). To evaluate neutrophil anti-bacterial function, we also analyzed neutrophil granule enzymes by qRT-PCR. We found that *Ot* infection resulted in a significant decrease of myeloperoxidase (MPO), which is known to be the key neutrophil component for bacterial killing (43, 44). In addition, the levels of proteinase-1 and elastase, which are neutrophil serine proteases for intracellular killing of pathogens, were comparable between mock and infected neutrophils (**Figure 2C**). Our *in vitro* data suggest that *Ot* may induce neutrophil activation and apoptosis, but inhibit MPO expression.

To further confirm that *Ot* can induce neutrophil apoptosis, we i.d. infected mice with CFSE-labeled bacteria in the ears and harvested the skin tissues for flow cytometric analysis at 18 h. As shown in **Figure 2D**, there was a significant increase in the percentages of CD45^+^Ly6G^+^ neutrophils that infiltrated the infected mouse ears as compared to the mock ears (10% vs. 5%, *p*<0.001). The result of apoptotic cell staining showed that neutrophils from infected mice exhibited more than a 5-fold increase of Annexin V levels (55% vs. 10%, *p*<0.001, **Figure 2D**), indicating that *Ot* induced extensive cell apoptosis at the inoculation site. To examine whether neutrophil death was attributed to *Ot* infection, we analyzed the Annexin V expression on CFSE^+^ (infected) and CFSE^-^ (uninfected) neutrophils. As shown in the last panel of **Figure 2D**, more than 55% of neutrophils were CFSE^+^ and Annexin V^+^, while only 10% of cells were CFSE^-^ but Annexin V^+^, indicating that most of *Ot*-infected neutrophils were undergoing apoptosis. These lines of evidence collectively argue that *Ot* infection triggers neutrophil activation and apoptosis in the skin.

### Neutrophils played a dispensable role for bacterial control in intradermal mouse model of *Ot* infection

**Figure 3:**
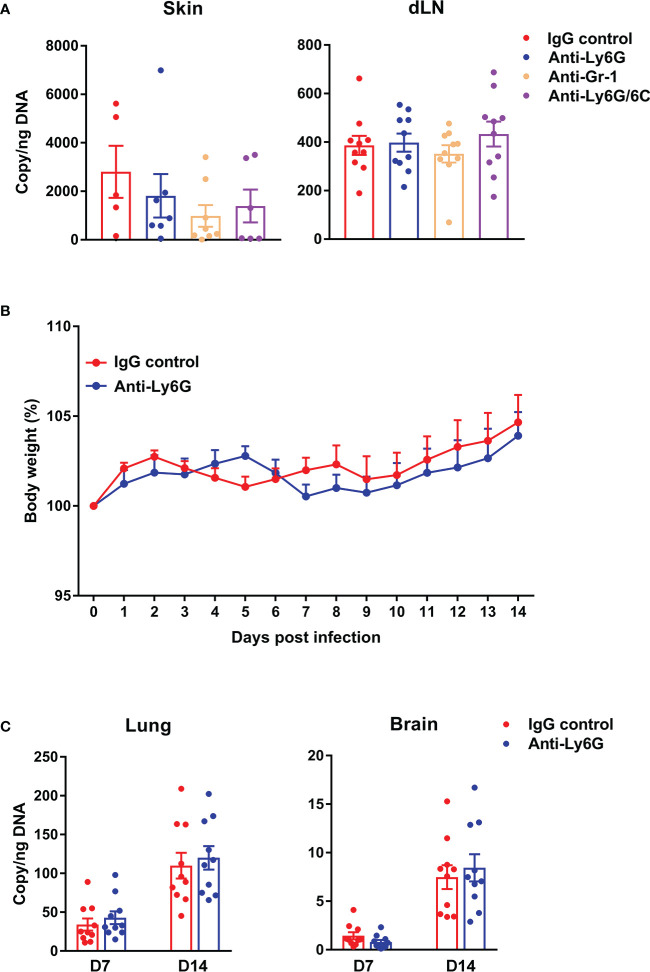
Depletion of neutrophils did not impact bacterial load in central or peripheral tissues of *Ot*-infected mice. B6 mice (n=10/group) were i.p. injected with anti-Ly6G, anti-Gr-1 or anti-Ly6G/6C antibody (clone#1A8, RB6-8C5 and NIMP-R14, respectively; 250 µg/mouse) at day -1, 0, 1 and 2 post-infection. Control mice were treated with IgG (250 µg/mouse). At day 0, mice were i.d. infected with *Ot* (4×10^3^ FFU) in the ears. **(A)** On day 3, skin tissue and draining lymph nodes (dLN) were collected for quantification of bacterial load. **(B)** Bodyweight following *Ot* infection. **(C)** Lung and brain tissues were collected at days 7 and 14 and bacterial load was quantified. Values are shown as mean ± SEM from single experiments and are representative of two independent experiments. A one-way ANOVA with a Tukey’s multiple comparisons test is used for statistical analysis. A two-tailed Student’s t-test was used for comparison of two groups. No significant difference was found in these results.

Having demonstrated neutrophil recruitment to the inoculation site to engulf bacteria and some neutrophil death (**Figures 1**, **2**), we next examined whether neutrophils could promote *Ot* dissemination *via* antibody-mediated cell depletion. We i.d. infected mice with *Ot* or PBS in the ears and i.p. treated mice with anti-Ly6G, anti-Gr-1, or anti-Ly6G/6C Abs at day -1, 0, 1 and 2 post-infection. Flow cytometric result confirmed the complete depletion of neutrophils in the skin following the antibody treatment (**Figure S2**). At day 3, we found comparable bacterial copy numbers in the skin and draining lymph nodes (**Figure 3A**), as well as comparable body weight changes (**Figure 3B**), between IgG control and antibody-treated groups. We also measured bacterial loads of mouse lung and brain tissues harvested at day 7 and 14 post-infection, and found comparable bacterial burdens in mice with or without neutrophil depletion (**Figure 3C**). In all, our data indicate a dispensable role of neutrophils for bacterial control and dissemination in our i.d. model of *Ot* infection.

### DCs facilitated bacterial dissemination from the inoculation site to the draining lymph nodes

**Figure 4:**
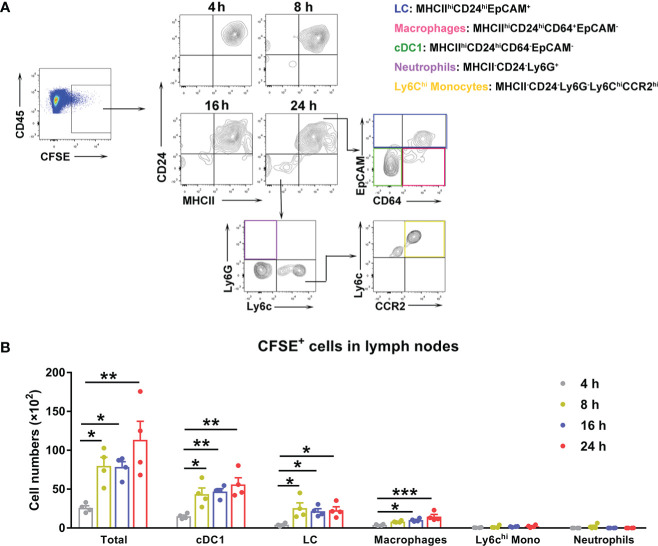
*Ot* infected dendritic cells and macrophages, which migrated to draining lymph nodes in mice. B6 mice (n=4/group) were i.d. infected with CFSE-labeled *Ot* (4×10^3^ FFU) in the ears. dLN were harvested at indicated time-points, and prepared for single cell suspension, followed by flow cytometric analysis. **(A)** Live cells were sorted by live/dead dye staining, and *Ot*-infected immune cells were identified by expression of CD45^+^CFSE^+^. We determined that *Ot* infected the following immune cell subpopulations: Langerhans cells (MHCII^hi^CD24^hi^EpCAM^+^), macrophages (MHCII^+^CD24^hi^CD64^+^EpCAM^-^), conventional dendritic cells (MHCII^hi^CD24^hi^CD64^-^EpCAM^-^), neutrophils (MHCII^-^CD24^-^Ly6G^+^), and Ly6C^hi^ monocytes (MHCII^-^CD24^-^Ly6G^-^Ly6C^hi^CCR2^hi^). **(B)** The absolute *Ot*-infected cell numbers in dLN were calculated. Values are shown as mean ± SEM from single experiments and are representative of two independent experiments. A one-way ANOVA with Tukey’s multiple comparisons test was used for statistical analysis. *, *p*<0.05; **, *p*<0.01; ***, *p <*0.001.

To determine the cell types that were relevant to bacterial dissemination, we i.d. injected mouse ears with CFSE-labeled *Ot* and harvested the cervical lymph nodes at 4, 8, 16 and 24 h for flow cytometric analysis, using our gating strategy shown in **Figure 4A** (45). The *Ot*-infected immune cells were gated as CD45^+^CFSE^+^ first, followed by the cell subpopulation gating as Langerhans cells (MHCII^hi^CD24^hi^EpCAM^+^), macrophages (MHCII^hi^CD24^hi^CD64^+^EpCAM^-^), cDC1 (MHCII^hi^CD24^hi^CD64^-^EpCAM^-^), neutrophils (MHCII^-^CD24^-^Ly6G^+^), and Ly6C^hi^ monocytes (MHCII^-^CD24^-^Ly6G^-^Ly6C^hi^CCR2^hi^), respectively. We found that *Ot*-infected cells were detected in dLN as early as 4 h, and gradually increased during 24 h post infection (**Figure 4B**). Importantly, most *Ot*-infected immune cells in dLN were cDC1 and LC, as well as some macrophages, suggesting that DCs and macrophages were the Trojan horse for bacterial dissemination from the inoculation site to dLN. CFSE^+^ neutrophils and Ly6C^hi^ monocytes were nearly undetectable, indicating that these cells were not the main carriers of *Ot* for dissemination to dLN (**Figure 4B**). Instead, DCs may play a key role in bacterial dissemination *via* transmission of *Ot* into dLN. We also infected bone marrow-derived DCs with *Ot in vitro* and analyzed cell activation markers. *Ot* infection only slightly increased CD86 expression, but not MHCII and CD80 at 24 and 48 h (**Figure S3A**). Moreover, we found that *Ot* replicated in DCs as evidenced by significantly increased bacterial burdens in the cultures (**Figure S3B**). To confirm that DCs can carry live *Ot* for migration into dLN, we purified CD11c^+^ cells from mouse dLN at day 2 post-infection. Cells (2 × 10^6^) were homogenized by glass beads, and the cell lysates were inoculated into L929 cells. At day 14 of cultivation, we found 6 × 10^4^ and 2 × 10^4^ copy numbers of *Ot* in L929 cells by the inoculation of CD11c^+^ and CD11c^-^ cell lysates, respectively (**Figure S4**). Bacteria were undetectable in uninfected L929 cells. Our results suggest that DCs might be the potential reservoirs of *Ot* and facilitate bacterial dissemination *in vivo*.

### Early bacterial dissemination was mediated *via* CCR7

**Figure 5:**
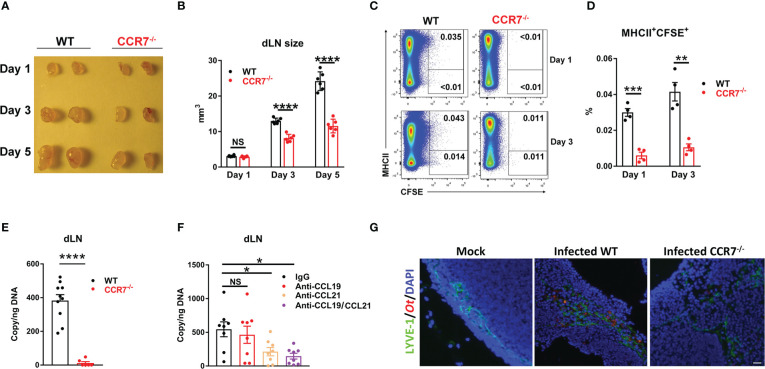
CCR7 knockout resulted in reduced bacterial dissemination into draining lymph nodes in mice. WT and CCR7^-/-^ mice (n=4-6/group) were i.d. infected with CFSE-labeled *Ot* (4×10^3^ FFU) in the ears. **(A)** dLN were harvested at indicated time-points and **(B)** the sizes of dLN were quantified. **(C, D)** Single cell suspension was prepared from dLN at days 1 and 3, followed by the flow cytometric analysis of *Ot*-infected dendritic cells (defined as MHCII^+^CFSE^+^). **(E)** Bacterial loads in dLN of WT and CCR7^-/-^ mice (n=10/group) were quantified at day 3 by qPCR. **(F)** B6 WT mice (n=8/group) were i.d. injected with anti-CCL19 (2 µg), anti-CCL21 (2 µg) or both (2 µg/each, 4 µg total) at 1 day prior to infection. Goat IgG was used as a control or supplemented to reach equal amount of antibody (4 µg total) per injection. Mice were infected with CFSE-labeled *Ot* (4×10^3^ FFU) in the ears and dLN were harvested at day 3 for bacterial load quantification. **(G)** dLN were collected from *Ot*-infected mice at day 5 and were processed for immunofluorescent staining. dLN tissue was stained for lymphatic endothelial cells (LYVE1, green), *Orientia* (TSA56, red) and DAPI (blue). Images were acquired by an Olympus IX73 inverted microscope. The scale bar is 20 µm. A one-way ANOVA with a Tukey’s multiple comparisons test is used for statistical analysis. A two-tailed Student’s t-test is used for comparison of two groups. *, *p*<0.05; **, *p*<0.01; ***, *p <*0.001; ****, *p* < 0.0001; NS, no significant difference.

The chemokine receptor CCR7 is known to govern skin DC migration and play a critical role in the entry of dermal and epidermal DCs into lymphatic vessels (46, 47). Our findings of DCs as the major cell populations for *Ot* transmission into the dLN (**Figure 4**) led us to speculate that the lack of CCR7 signaling may subsequently reduce early bacterial dissemination. To test this hypothesis, we i.d. infected WT and CCR7^-/-^ mice with CFSE-labeled *Ot* and harvested the dLN at days 1, 3 and 5. The CCR7 deficiency resulted in decreased size of the dLN at days 3 and 5 when compared to WT controls (**Figures 5A, B**). Flow cytometric results demonstrated less *Ot*-carrying DCs in the dLN in the absence of CCR7 as evidenced by decreased MHCII^+^CFSE^+^ cells in CCR7^-/-^ mice at days 1 and 3 (**Figures 5C, D**). Moreover, the bacterial copy numbers of the dLN were much lower in CCR7^-/-^ mice (**Figure 5E**), indicating that CCR7 mediated bacterial dissemination from the inoculation site into the dLN. Since CCL19 and CCL21 are the unique ligands for CCR7, contributing to DC migration into the dLN, we treated mice with CCL19- and CCL21-neutralizing Abs prior to infection and subsequently measured bacterial burdens in dLN. Our findings showed that the dLN of anti-CCL21-treated mice had significantly lower bacterial burdens as compared to mice received control IgG (**Figure 5F**). Given that *Ot*-infected DCs are known to have the ability to migrate from inoculation site into dLN *via* lymphatic vessels (14), we used fluorescent microscopy and found less *Ot* (red) in the dLN of CCR7^-/-^ mice at day 3 as compared to that of WT mice. Moreover, most bacterial staining was clustered in LYVE-1^+^ (green) lymphatic vessels (**Figure 5G**), indicative of early endothelial infection in dLN by *Ot* bacteria.

## Discussion

*Ot* bacteria are transmitted to humans by the bite of infected chiggers (larval mites) and can cause severe illness, multi-organ failure and patient death (2, 3). Early innate immunity in the inoculation site is the first line of defense against invading pathogens; however, little is known about the local immune responses against infection, or the mechanism by which *Ot* disseminates from the inoculation site (skin) to visceral organs. In this study, we i.d. infected C57BL/6j mice with CFSE-labeled *Ot* and depicted the dynamic pattern of skin immune cell responses by multicolor flow cytometry. We found that neutrophils infiltrated in the inoculation site quickly, followed by monocytes, Langerhans cells and cDC1. *Ot* infection increased neutrophil inflammatory molecule expression and promoted neutrophil apoptosis both *in vitro* and *in vivo*. However, neutrophil depletion did not change tissue bacterial loads, indicating that neutrophils may not play a decisive role in our i.d. mouse model of *Ot* infection. Further analyses demonstrated that DCs and macrophages were the major *Ot*-carrier for bacterial dissemination from the skin inoculation site to the dLN. Loss of CCR7, a key chemokine receptor for dendritic cell lymph node homing, resulted in significant reduction of *Ot*-carrying DCs and bacterial loads in the dLN. By using neutralizing antibodies, we also found that CCL21, but not CCL19, played a critical role for bacterial dissemination into dLN. Interestingly, it is reported that CCL21, but not CCL19, is highly expressed by endothelial cells in lymph nodes (48). Our result suggests that *Ot* infection may induce CCL21 production in lymphatic endothelial cells and promote the migration of *Ot*-infected myeloid cells, leading to the accelerated bacterial dissemination from the skin to dLN. Therefore, we revealed a novel mechanism of *Ot* dissemination through a CCR7/CCL21-dependent way.

To investigate the host immune responses against *Ot* infection, several types of animal models have been established (24). Although the chigger-infected animal models most accurately mimic human infection by chigger bites, it is not available to most research laboratories due to the lack of infected chiggers (24). Instead, we have established an i.d. murine model of *Ot* infection that elicited systemic inflammatory responses and histopathological changes among major organs during acute stages, indicating that i.d. infection in part, mimics natural infection in humans (28). Unlike intraperitoneal or intravenous inoculation that cause lethal infection in mice, i.d. infection of *Ot* in mice is non-lethal, triggering only 5-10% bodyweight loss at day 10-11 post-infection, followed by full recovery (28). This clinical course is reminiscent of most patients during natural infection and well-suited for studying early immune activity and bacterial dissemination in the host. Interestingly, i.d. infection in humanized mice can result in animal lethality (20), suggesting that diversity of host immunity is critical for disease outcome of scrub typhus.

Tracking pathogens’ tissue tropism *in vivo* is an important aspect of investigating organism dissemination and host/pathogen interactions. Currently, there is no genetic tool available for *Ot* because of its sensitivity to osmotic pressure, and difficulty to genetically modify it due to massive amplification of repetitive sequences in its genome (29). To overcome this challenge, fluorescent probes have been used to label and track *Ot* for live cell imaging by fluorescence microscopy (30). Accordingly, we utilized CFSE-labeled *Ot* for i.d. infection in mice and analyzed skin cell samples by multicolor flow cytometry. Notably, we only assessed the CFSE-labeled cells within 24 h post-infection to avoid significant reductions of CFSE fluorescence *in vivo* [28]. Although lymphocytes (e.g., αβ T, γδ T, NKT, NK and ILC) may also migrate into the inoculation site, they could not be the cell targets of *Ot* invasion (10). It is known that *Ot* mainly infects endothelial cells in the peripheral organs; however, myeloid cells (e.g., DCs, macrophages, neutrophils and monocytes) are considered the major target of *Ot* at the inoculation site (10, 49, 50). *Ot*-infected myeloid cells could enhance antibacterial activity, contributing to bacterial control through phagocytosis, antimicrobial enzymes, reactive oxygen species (ROS) and extracellular traps (51). Moreover, *Ot* can trigger inflammasome activation and induce chemokine production in macrophages, resulting in recruitment of other immune cells, such as neutrophils, monocytes and T cells (52, 53). We also recently identified Mincle as a key C-type lectin receptor for *Ot* infection, and the subsequent inflammatory mediator expression in macrophages (54). In addition to their antibacterial and proinflammatory roles, we demonstrated in this study that DCs and macrophages may act as carriers of *Ot*, and facilitate bacterial dissemination into dLN and peripheral organs (**Figures 4**, **5**). However, the key components that regulate antimicrobial responses and myeloid cell migration remain unclear and need further study.

Neutrophils are potent phagocytes and the first line of defense following infection; however, they can also serve as the Trojan horse in pathogen transmission from the initial bite site to visceral organs. Several microorganisms (e.g., *Leishmania*, *Chlamydia pneumoniae*, and *Yersinia pestis*) have developed capabilities to invade neutrophils and avoid neutrophil-mediated killing, or to take advantage of neutrophil clearance pathways, such as efferocytosis, to promote their dissemination in the host (55, 56). However, the role of neutrophils in bacterial dissemination of *Ot* infection is not well defined. Our results showed that although neutrophils uptake *Ot* rapidly and efficiently at the inoculation site, very few *Ot*-carrying neutrophils were detected in the dLN. Instead, DCs and macrophages were the major cell populations for *Ot* dissemination (**Figure 4**). Further analysis, both *in vitro* and *in vivo*, indicated that *Ot*-infected neutrophils significantly elevated the apoptosis marker Annexin V, and their fate tended toward cell death (**Figure 2**). The *in vivo* depletion of neutrophils *via* neutralizing antibodies against Ly6G resulted in comparable bacterial loads in the skin and dLN at early infection as well as in the lung and brain at late stages. These results suggest a dispensable role of neutrophils in both early bacterial dissemination and the subsequent systemic infection. A potential reason for this finding could be attributed to the high level of neutrophil death and the downregulation of antimicrobial MPO by *Ot* infection (**Figure 2**). One limitation of this experiment is that our inoculation dose might result in saturated infection in the skin that masks the role of neutrophils during early infection. However, the accurate dose of *Ot* infection in human patients is still unknown. Additionally, it could be hard to track CFSE^+^ cells *in vivo* by i.d. infecting animals with a low dose of *Ot*. Another remaining question is whether dead neutrophils were phagocytized by efferocytosis and assisted DCs for bacterial migration. Further study by using genetically manipulated *Ot* or deep imaging of live tissue may improve our knowledge of *Ot* dissemination and bacteria/host immune cell interaction during early infection.

Although *Ot* mainly infects endothelial cells in visceral organs, eschar skin biopsies from patients with scrub typhus displayed an *Ot* tropism for DCs and monocytes (10), indicating that *Ot* may trigger distinct cell populations based on the stages of infection. Choi et al., reported that *Ot* escapes autophagy in DCs and upregulates the expression of CCR7, a key chemokine receptor for DC migration (14). Interestingly, DCs with *in vitro Ot*-infection displayed comparable migratory capacity for entry into lymph nodes when compared to unstimulated DCs, indicating that *Ot* may not induce dendritic cell maturation as strongly as LPS (14). Instead of infecting DCs *in vitro*, we directly i.d. injected CFSE-labeled bacteria in the ears and tracked the infected DCs (MHCII^+^CFSE^+^) *in vivo* (**Figure 5**). The regional lymphadenopathy in WT mice was observed as early as day 3 and became more apparent at day 5; however, the sizes of dLN were smaller in the absence of CCR7 as compared to the WT mice (**Figures 5A, B**). Importantly, the percentages of CFSE^+^ DCs were significantly lower in CCR7^-/-^ mice, suggesting that absence of CCR7 reduces the number of *Ot*-carrying DCs that can migrate into the dLN (**Figures 5C, D**). Our finding of diminished bacterial loads in the dLN of CCR7^-/-^ mice confirmed our conclusion that CCR7 plays a key role in *Ot* dissemination (**Figure 5E**). To affirm the key role of CCR7 signaling in early bacterial dissemination, we used neutralizing antibodies to block CCL19 and CCL21, as both are CCR7 ligands. Our data showed that blockage of CCL21, but not CCL19, resulted in reduction of bacterial loads in the dLN, suggesting that the CCR7/CCL21 axis plays an essential role for *Ot* dissemination (**Figure 5F**). In addition to DCs, M1 macrophage chemotaxis is also mediated by CCR7 (57). Given that *Ot* can infect macrophages and promote M1 polarization *in vivo* (23, 58), deficiency of CCR7 may also impair the migration of *Ot*-carrying M1 macrophages into dLN. As shown in **Figure 4B**, we indeed found some CFSE^+^ macrophages in the lymph nodes. However, such data may reflect different possibilities: 1) local capture of free or released *Ot* by macrophages; 2) *Ot*-carrying monocytes/macrophages migrate from the skin into dLN. The exact role of DCs and macrophages in bacterial dissemination needs further investigation. To investigate the process and cellular target of *Ot* infection in regional LNs, we analyzed tissue by fluorescent staining and microscopy. Our images clearly showed the localization of *Ot* within the lymphatic vessels, demonstrating that *Ot* has the tropism for lymphatic endothelial cells in the lymph nodes at early stage of infection.

**Figure 6:**
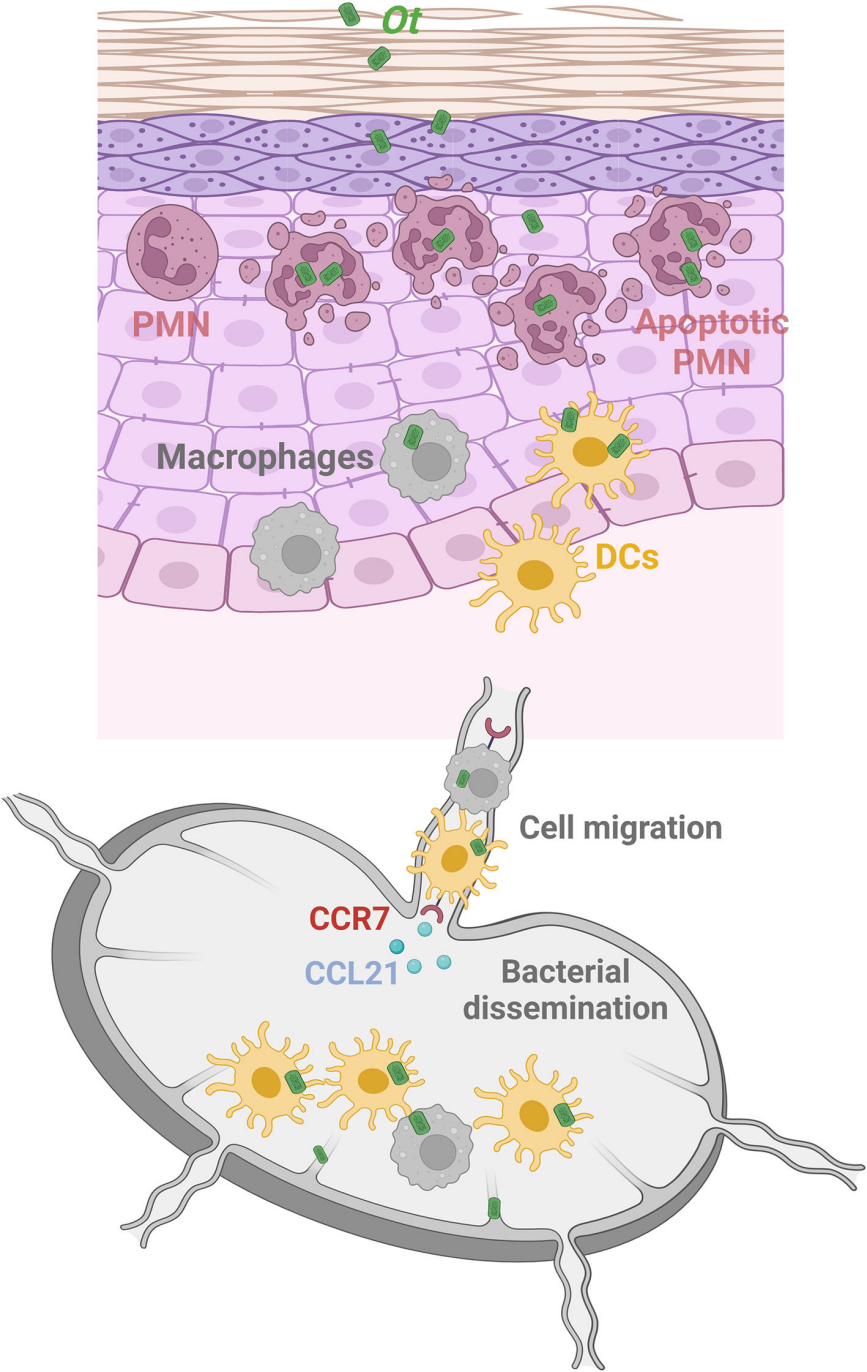
Graphical illustration of *Ot* dissemination and innate immune cell responses in an i.d. infection mouse model. Following i.d. infection with *Ot*, neutrophils are the first responders and rapidly infiltrate the skin. Although neutrophils (purple) efficiently engulf bacteria in the inoculation site, *Ot* induces neutrophil apoptosis locally, and neutrophils cannot carry *Ot* into the dLN. Instead, DCs (yellow) and macrophages (gray), which also migrate into the skin for bacterial uptake, are the reservoirs responsible for bacterial dissemination of *Ot* to the dLN. Notably, they may serve as “Trojan horse” vehicles that carry *Ot* and migrate into the dLN in a CCR7/CCL21-dependent way to facilitate bacterial replication and dissemination. In addition, lymphatic vessels are also infected by *Ot*, indicating that lymphatic ECs could be the target cells of *Ot* replication in the dLN for further bacterial dissemination into visceral organs. This graphical illustration is created with BioRender.com.

In conclusion, our study has delineated the dynamic profile of immune cell responses within the *Ot*-inoculation site and revealed the CCR7/dendritic cell axis as a major component of early bacterial dissemination (**Figure 6**). Further investigation is warranted to identify the key anti-bacterial effector molecules and the underlying mechanism(s) of early innate immunity against *Ot* dissemination and growth. Such findings may provide novel insight into the efficient vaccine development strategies for scrub typhus as well as other intracellular bacterial infection.

## Data availability statement

The raw data supporting the conclusions of this article will be made available by the authors, without undue reservation.

## Ethics statement

The animal study was reviewed and approved by Mice were maintained under specific pathogen-free conditions and used at 8-10 weeks of age, following protocols approved by the Institutional Animal Care and Use Committee (protocol # 2101001) at the University of Texas Medical Branch (UTMB) in Galveston, TX. All mouse infection studies were performed in the ABSL3 facility in the Galveston National Laboratory located at UTMB; all tissue processing and analysis procedures were performed in the BSL3 or BSL2 facilities. All procedures were approved by the Institutional Biosafety Committee, in accordance with Guidelines for Biosafety in Microbiological and Biomedical Laboratories. UTMB operates to comply with the USDA Animal Welfare Act (Public Law 89–544), the Health Research Extension Act of 1985 (Public Law 99–158), the Public Health Service Policy on Humane Care and Use of Laboratory Animals, and the NAS Guide for the Care and Use of Laboratory Animals (ISBN-13). UTMB is a registered Research Facility under the Animal Welfare Act, and has a current assurance on file with the Office of Laboratory Animal Welfare, in compliance with NIH Policy.

## Author contributions

YL and LS contributed to conception and design of the study. YL, HW, CG and JT performed experiments, data collection and statistical analysis. YL wrote the first draft of the manuscript. HW, CG, JT, PS, JS, PM and LS, revised the manuscript. All authors contributed to the article and approved the submitted version.
