# Supplementary material for: CCR7/dendritic cell axis mediates early bacterial dissemination in Orientia tsutsugamushi-infected mice
Source: Front Immunol. 2022 Dec 22;13:1061031. doi: 10.3389/fimmu.2022.1061031 (PMC9813216; doi:10.3389/fimmu.2022.1061031)
Supplement: Supplementary file 1 [file DataSheet_1.docx]

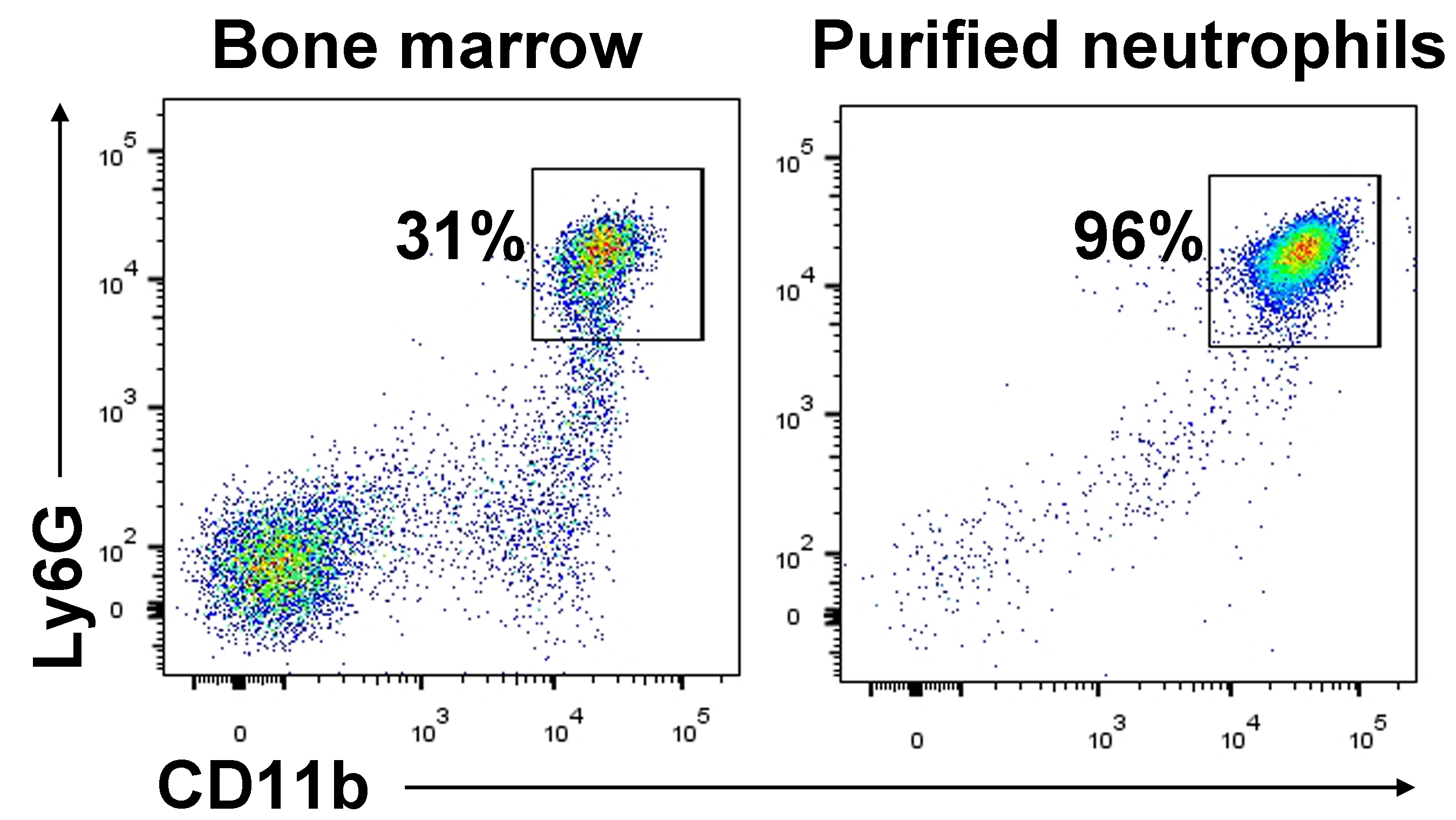


**Figure S1.** **The purity of bone marrow-derived neutrophils.** Bone marrow cells were collected from the tibia and femur of B6 mice, followed by incubation with Red Cell Lysis Buffer buffer for 5 mins at room temperature to remove red blood cells. Neutrophils were purified by anti-Ly6G magnetic beads in LS column of positive selection. The purity of neutrophils were determined by flow cytometry.


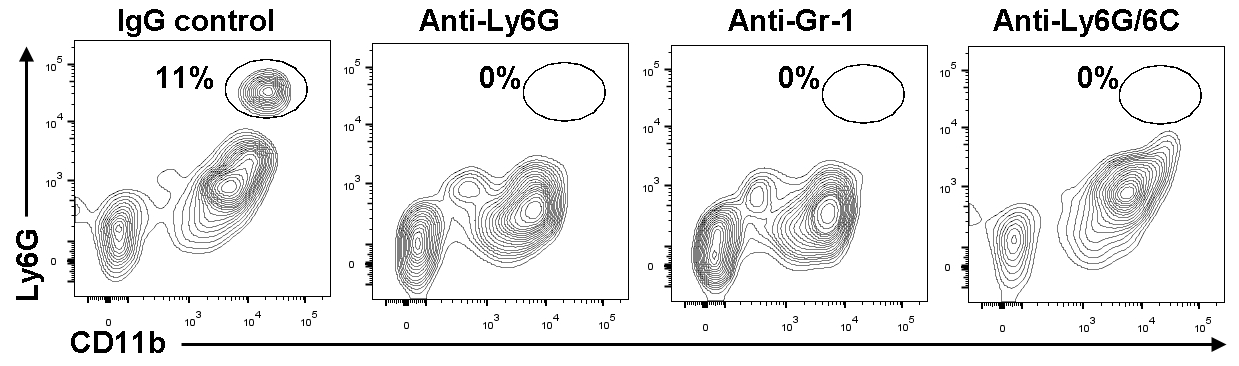


**Figure S2. The depletion of neutrophils in the skin.** B6 (3/group) mice were i.p. injected with anti-Ly6G, anti-Gr-1 or anti-Ly6G/6C (clone#1A8, RB6-8C5 and NIMP-R14, respectively; 250 µg/mouse) at one day prior to infection. Control mice were treated with IgG (250 µg/mouse). At day 0, mice were i.d. infected with *Ot* (4×10^3^ FFU) in the ears. At 24 h post infection, skin tissues were harvested for neutrophil detection by flow cytometry.


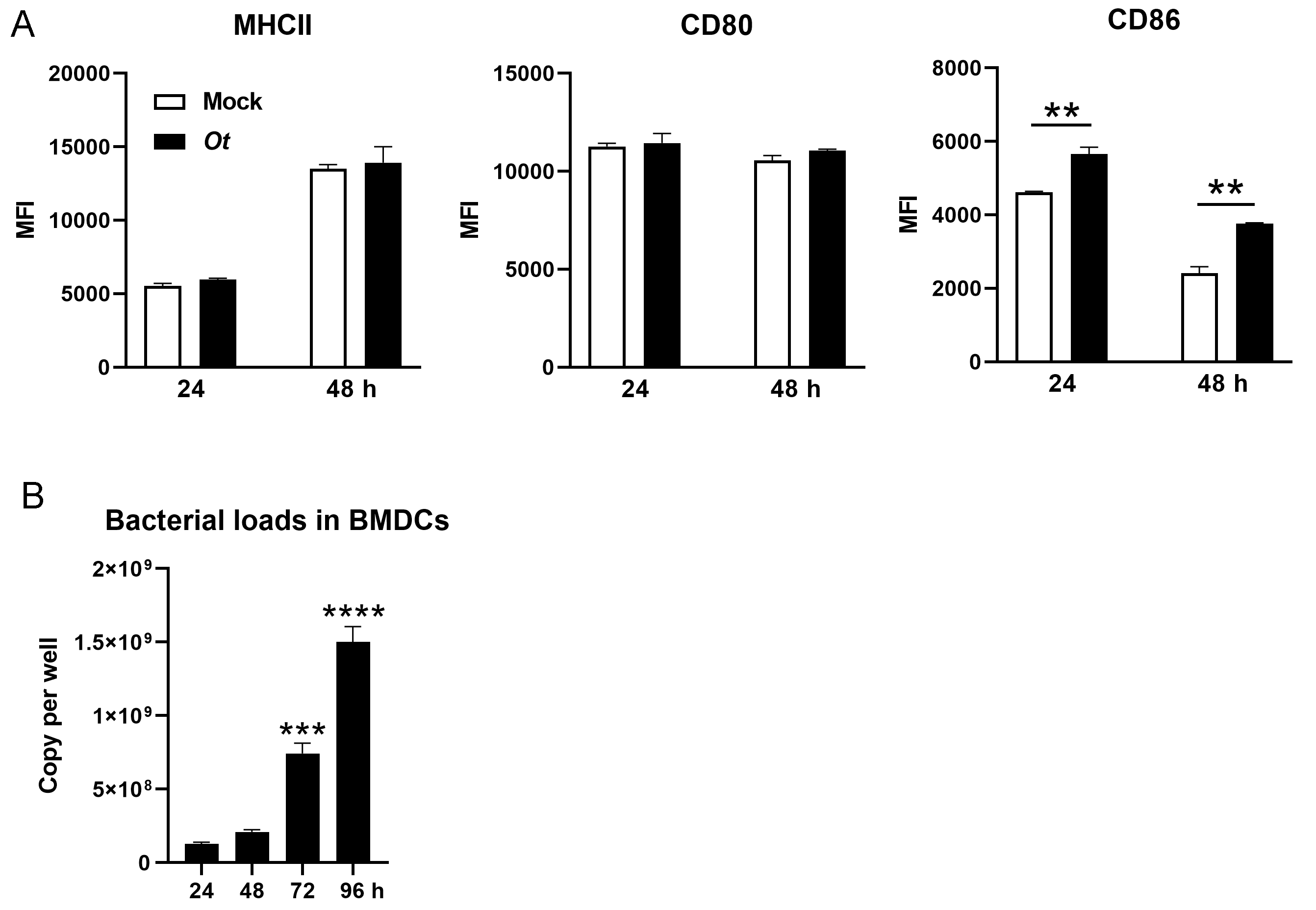


**Figure S3. DC activation by *Ot* infection.** Bone marrow-derived DCs were seeded in 12 well plates, followed by *Ot* infection (MOI 10). (A) Cells were harvested at 24 and 48 h post-infection and analyzed by flow cytometry. The mean fluorescence intensity of MHCII, CD80 and CD86 were shown. (B) Bacterial loads were measured at 24, 48, 72 and 96 h post-infection by qPCR. The 24 h group was used as a control for comparison. Each group contains three samples. Values are shown as mean ± SEM from single experiments and are representative of two independent experiments. A two-tailed student t test is used for comparison of two groups. A one-way ANOVA with a Tukey’s multiple comparisons test is used for statistical analysis. **, *p*<0.01; ***, *p* <0.001; ****, *p* <0.0001.


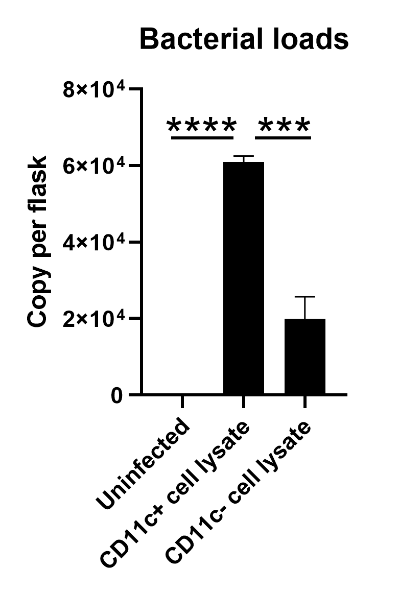


**Figure S4. DC carried live *Ot* and migrated into dLN.** B6 mice were i.d. infected with *Ot* (4×10^3^ FFU) in the ears and dLN were harvested for cell purification. DCs were purified by using anti-CD11c magnetic beads and positive selection. CD11c- cells were also collected for a control. Cells (2 × 10^6^) were homogenized by glass beads and the cell lysates were inoculated into L929 cells. At day 14 of culture, we measured bacterial loads in all wells of cell culture plates by qPCR. A one-way ANOVA with a Tukey’s multiple comparisons test is used for statistical analysis. ***, *p* <0.001; ****, *p* <0.0001.
