# Supplementary material for: CCR7/dendritic cell axis mediates early bacterial dissemination in Orientia tsutsugamushi-infected mice
Source: Front Immunol. 2022 Dec 22;13:1061031. doi: 10.3389/fimmu.2022.1061031 (PMC9813216; doi:10.3389/fimmu.2022.1061031)
Supplement: Supplementary file 2 [file Table_1.docx]

| **Supplementary table 1** | | | |
| --- | --- | --- | --- |
|  |  |  |  |

**Real-time PCR primers of murine genes**

Forward (5’ to 3’) Reverse (5’ to 3’)

ICAM1 GTGATGCTCAGGTATCCATCCA CACAGTTCTCAAAGCACAGCG

TNF-α CCCTCACACTCAGATCATCTTCT GCTACGACGTGGGCTACAG

Arg1 CTCCAAGCCAAAGTCCTTAGAG AGGAGCTGTCATTAGGGACATC

CASP3 ATGGAGAACAACAAAACCTCAGT TTGCTCCCATGTATGGTCTTTAC

CXCL10 CCAAGTGCTGCCGTCATTTTC GGCTCGCAGGGATGATTTCAA

MPO AGTTGTGCTGAGCTGTATGGA CGGCTGCTTGAAGTAAAACAGG

Proteinase1 TTCAGCCCTTGCTTGCCTC ACACTTTTACTCCGAAGTCGGT

Elastase AGCAGTCCATTGTGTGAACGG CACAGCCTCCTCGGATGAAG

iNOS GTTCTCAGCCCAACAATACAAGA GTGGACGGGTCG ATGTCAC

GAPDH TGGAAAGCTGTGGCGTGAT TGCTTCACCACCTTCTTGAT
